# Chondroitin Sulfate‐Coated Heteroduplex‐Molecular Spherical Nucleic Acids

**DOI:** 10.1002/cbic.202400908

**Published:** 2024-11-28

**Authors:** Toni Laine, Prasannakumar Deshpande, Ville Tähtinen, Eleanor T. Coffey, Pasi Virta

**Affiliations:** ^1^ Department of Chemistry University of Turku 20500 Turku Finland; ^2^ Turku Bioscience Centre University of Turku, Åbo Akademi University 20520 Turku Finland

**Keywords:** Molecular Spherical Nucleic Acids, Heteroduplex ASOs, Chondroitin sulfates, Nanoparticles, Drug Delivery

## Abstract

Molecular Spherical Nucleic Acids (MSNAs) are atomically uniform dendritic nanostructures and potential delivery vehicles for oligonucleotides. The radial formulation combined with covalent conjugation may hide the oligonucleotide content and simultaneously enhance the role of appropriate conjugate groups on the outer sphere. The conjugate halo may be modulated to affect the delivery properties of the MSNAs. In the present study, [60]fullerene‐based molecular spherical nucleic acids, consisting of a 2′‐deoxyribonucleotide and a ribonucleotide sequence, were used as hybridization‐mediated carriers (“DNA and RNA‐carriers”) for an antisense oligonucleotide, suppressing Tau protein, (i. e. Tau‐ASO) and its conjugates with chondroitin sulfate tetrasaccharides (CS) with different sulfation patterns. The impact of the MSNA carriers, CS‐moieties on the conjugates and the CS‐decorations on the MSNAs on cellular uptake and ‐ activity (Tau‐suppression) of the Tau‐ASO was studied with hippocampal neurons *in vitro*. The formation and stability of these heteroduplex ASO‐MSNAs were evaluated by UV melting profile analysis, polyacrylamide gel electrophoresis (PAGE), dynamic light scattering (DLS) and size exclusion chromatography equipped with a multi angle light scattering detector (SEC‐MALS). The cellular uptake and ‐ activity were studied by confocal microscopy and Western blot analysis, respectively.

## Graphical abstract

[60]fullerene‐based molecular spherical nucleic acids (MSNAs) were used as hybridization‐mediated carriers for an antisense oligonucleotide, suppressing Tau protein, and its conjugates with chondroitin sulfate (CS) tetrasaccharides. The impact of the MSNA‐carriers and the CS‐halo on the cellular uptake and suppression of Tau‐protein was studied with hippocampal neurons *in vitro*.

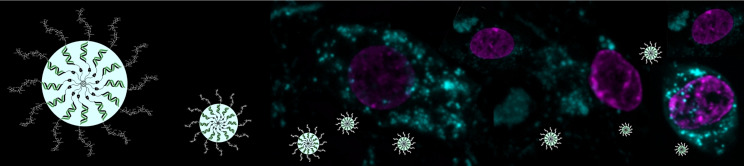

## Introduction

Spherical nucleic acids (SNAs, introduced by Mirkin's group) are nanostructural delivery vehicles, consisting of a dense shell of oligonucleotides attached to a suitable core unit.[^1^, ^2^] The core structure can be for example gold,[^1^, ^3^] silica,^[4]^ liposomes^[5]^ or proteins.^[6]^ SNAs may undergo class A scavenger receptor–mediated endocytosis,[^7^, ^8^] which has been demonstrated by the successful free uptake of antisense oligonucleotides and siRNAs^[9]^ in more than 50 different cell lines.[^3^, ^10^] In addition, atomically uniform variants of SNAs, i. e. molecular spherical nucleic acids (MSNAs)[^11^, ^12^, ^13^, ^14^, ^15^, ^16^, ^17^, ^18^] and other dendritic oligonucleotides^[19]^ have received growing interest as delivery vehicles. The lower oligonucleotide density of MSNAs results in weaker activation of scavenger receptors,[^11^, ^20^] but MSNAs may become attractive delivery vehicles when they are conjugated with cell/tissue‐specific ligands. The radial formulation connected to covalent conjugation may hide the oligonucleotide content and simultaneously enhance the role of appropriate conjugate groups on the outer surface, which may be tuned to find enhanced precision delivery for oligonucleotides. Indeed, our recent *in vivo* PET‐imaging study^[15]^ shows that *i. v.‐*administrated MSNAs exhibit prolonged circulation times due to prevented accumulation in the kidney, and liver, in comparison to linear phosphorothioate oligonucleotides[^21^, ^22^] and most nanoparticular delivery vehicles,[^23^, ^24^] respectively. The biodistribution and targeted delivery could be further adjusted by the surface decoration (folate^[25]^ and trastuzumab^[12]^ demonstrated) of MSNAs.

**Figure 1:**
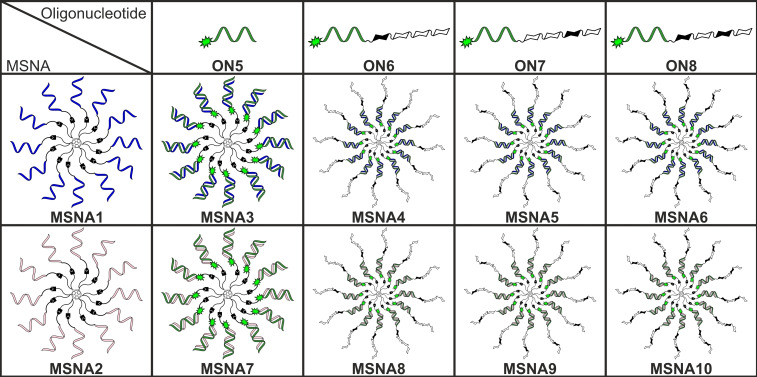
Illustration of the MSNA structures in the study. **MSNA1** and **MSNA2** are hybridized with **ON5**–**8** to yield **MSNA3**–**10** (cf. Scheme 1). Filled sugar symbols refer to sulfated *N*‐acetylgalactosamine units.

In the present study, [60]fullerene‐based MSNAs, consisting of 2′‐deoxy oligoribonucleotide (**MSNA1**) or oligoribonucleotide‐strands (**MSNA2**), were synthesized and used as hybridization‐mediated carriers (“DNA‐ and RNA‐carriers”) for an antisense oligonucleotide sequence, suppressing Tau protein, (i. e. Tau‐ASO, cf. **ON5**–**8**) (Figure 1). Tau is a protein expressed in neurons whose hyperphosphorylation and aggregation are important factors in neurodegeneration caused by several diseases, including Alzheimer's disease.[^26^, ^27^] Regulation of pathological Tau can prevent symptoms of Alzheimer's disease and restore lost function.[^28^, ^29^, ^30^] Antisense oligonucleotides (ASOs) targeting Tau protein have been developed,[^29^, ^31^, ^32^] including positive phase I trials in humans with mild Alzheimer's disease.^[31]^ Heteroduplex[^33^, ^34^] Tau‐ASOs are assembled on the MSNAs (**MSNA3**–**10**). On the DNA‐MSNA carrier (**MSNA1**), the release of the active Tau‐ASO (consisted of phosphorotioate backbone) can undergo passive dissociation or DNase‐mediated degradation of the carrier (consisted of native phosphodiester backbone). On the RNA‐MSNA (**MSNA2**), in turn, RNase H‐mediated degradation of the RNA‐based carrier may take place, releasing the active Tau‐ASO‐sequence. The impact of the MSNA carrier and its chemistry (DNA vs RNA, **MSNA3** vs **MSNA7**) on hippocampal neuron uptake and activity to suppress Tau protein were studied *in vitro* by confocal microscopy and Western blot analysis, respectively. In addition to MSNA formulation, the role of chondroitin sulfate tetrasaccharides with different sulfation patterns as surface decoration of MSNAs (**MSNA4**–**6** and **MSNA8**–**10**) to the hippocampal neuron uptake was examined. The glycosaminoglycan chondroitin sulfate (CS) is a linear polysaccharide important for several biological processes including cell division, cancer progression and metastasis, development of the central nervous system and central nervous system injuries and diseases.[^35^, ^36^, ^37^] The specific sulfation patterns determine the binding of CS to certain proteins such as neurotrophins, selectins, chemokines and midkine.[^38^, ^39^, ^40^, ^41^] The ability to interact with certain proteins makes CS a potential tool for drug delivery. In addition, CS proteoglycans have been found to bind to several neuronal receptors.[^42^, ^43^] Previously, it has been shown that coating gold nanoparticles with specific chondroitin sulfates can enhance their targeting and uptake in neuronal cells.^[44]^ MSNAs are particularly attractive scaffolds as they can present multiples of each ligand on their outer surface. This is especially useful with weakly binding ligands whose binding is multivalent. The binding of CS to some proteins has been found to be multivalent as well.[^45^, ^46^, ^47^] This has prompted to research on synthetic analogues, such as polymers[^48^, ^49^] and dendrimers,[^46^, ^50^, ^51^, ^52^] that present CS‐ligands on their outer surface in an attempt to mimic natural CS polysaccharides.

## Results and Discussion

### Synthesis of CS‐Oligonucleotide Conjugates

**Scheme 1:**
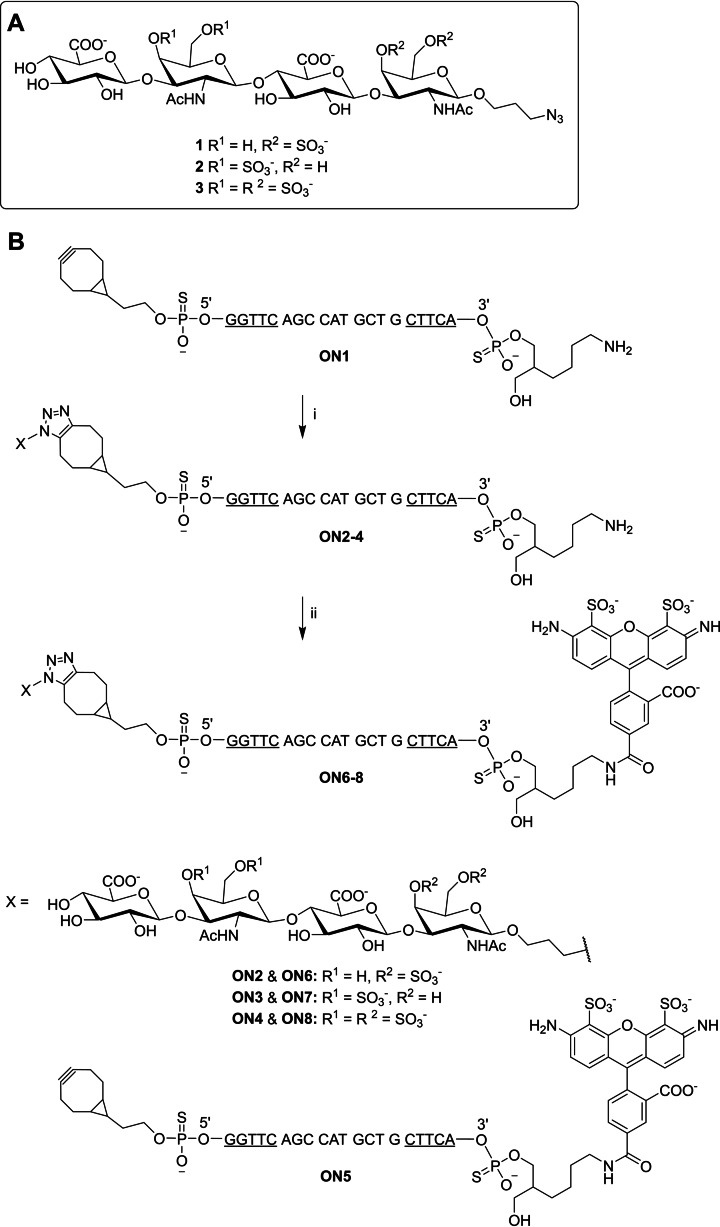
A) Structures of the azidopropyl‐modified CS tetrasaccharides.^[53]^ B) Synthesis of AF488‐labeled oligonucleotide conjugates **ON5**–**8**. The oligonucleotides are consisted of phosphorothioate backbone. Underlined letters represent 2′‐*O*‐(2‐methoxyethyl) ribonucleotides, the rest are 2′‐deoxyribonucleotides. Underlined C is 5‐methylated. Reagents and conditions: (i) 3 equivalents of azidopropyl‐modified CS tetrasaccharides **1**–**3**, H_2_O, overnight at room temperature. (ii) 20 equivalents of AF488 NHS ester, 0.1 M sodium borate (pH 8.4), H_2_O:DMSO (9 : 1, *v/v*), overnight at room temperature

The synthesis of the Alexa Fluor (AF488)‐labeled CS‐oligonucleotide conjugates is outlined in Scheme 1. 5′‐(1*R*,8*S*,9 *s*)‐Bicyclo[6.1.0]non‐4‐yn‐9‐yl (BCN) modified phosphorothioate oligonucleotide **ON1** was synthesized on a 1 μmol scale on a commercial amino‐modifier solid support using an automatic DNA/RNA synthesizer and commercially available phosphoramidite building blocks. **ON1** was conjugated with azidopropyl‐modified chondroitin sulfate (CS) tetrasaccharides^[53]^ **1**–**3** by strain‐promoted alkyne‐azide cycloaddition (SPAAC): **ON1** was incubated with compounds **1**–**3** (3 equiv; following previously published procedure^[53]^) overnight at room temperature. The resulting CS‐oligonucleotide conjugates (**ON2**–**4**) were homogenized by RP‐HPLC and authenticity of the products was verified by MS (ESI‐TOF) spectroscopy (Figures S1–S3 and Table S1). Isolated yields of **ON2**–**4** varied between 18 and 22 %. Oligonucleotides **ON1**–**4** were labelled with an AF488 fluorescent dye: AF488 NHS ester (in DMSO) was added into a solution of the oligonucleotide (in aqueous borate buffer, pH 8.4) and the reaction mixture was incubated overnight at room temperature. The resulting AF488‐labeled oligonucleotide conjugates (**ON5**–**8**) were purified by RP‐HPLC and authenticity of the products was confirmed by MS (orbitrap) spectroscopy (Figures S4–S7 and Table S1). Isolated yields of **ON5**–**8** varied between 85 % and 91 %.

### Synthesis of MSNAs

**Scheme 2:**
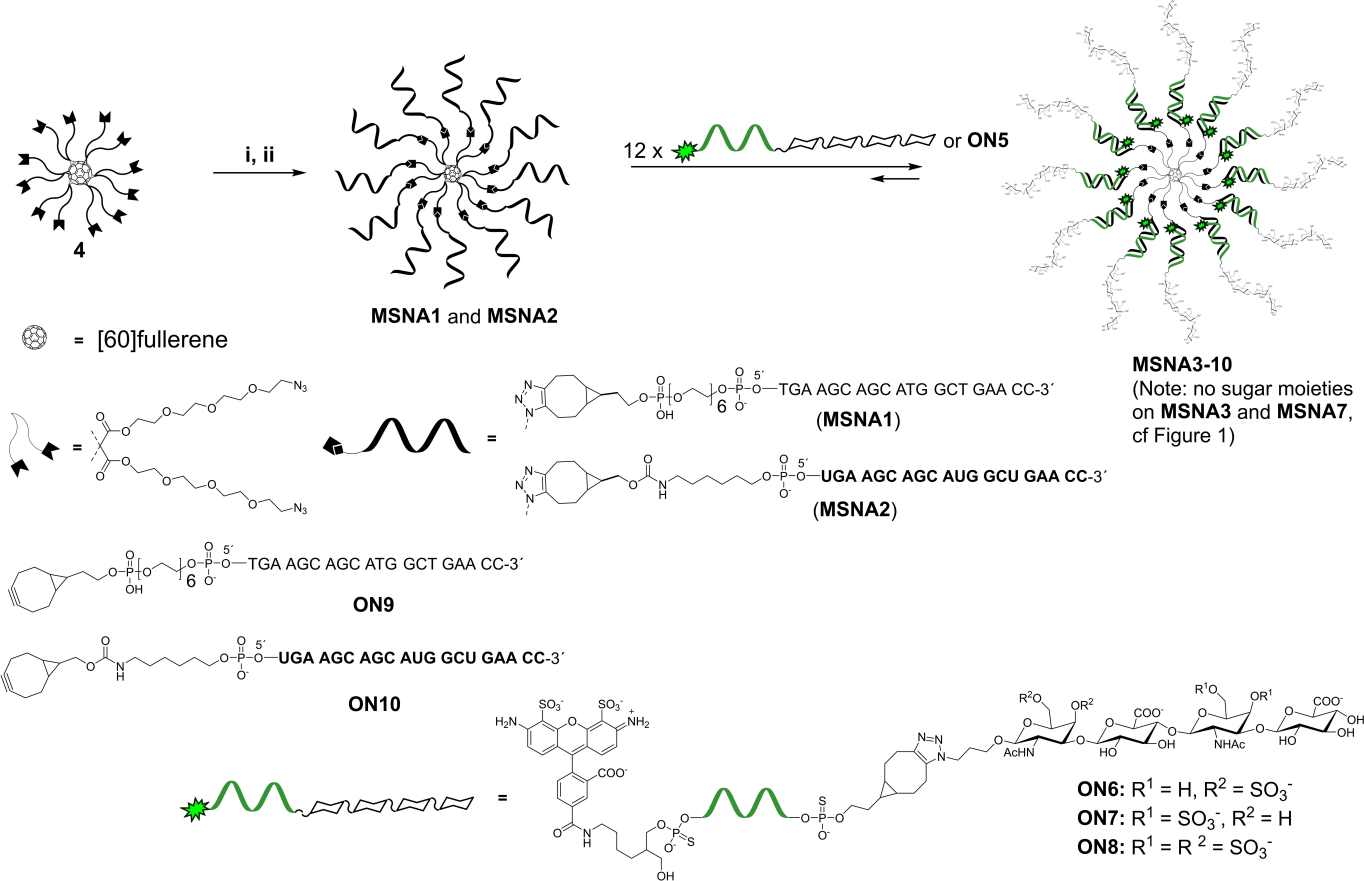
Synthesis and hybridization‐mediated assembly of MSNAs. Letters in bold represent ribonucleotides Conditions: i) **ON9** or **ON10** (0.3 equivalents/**4**), DMSO:H_2_O (9 : 1, *v/v*), overnight at room temperature; ii) **ON9** or **ON10** (1.2 or 1.5 equivalents / azide arm of **4**), 1.5 M or 0.75 M NaCl (aq), 3 days at room temperature.

**Figure 2:**
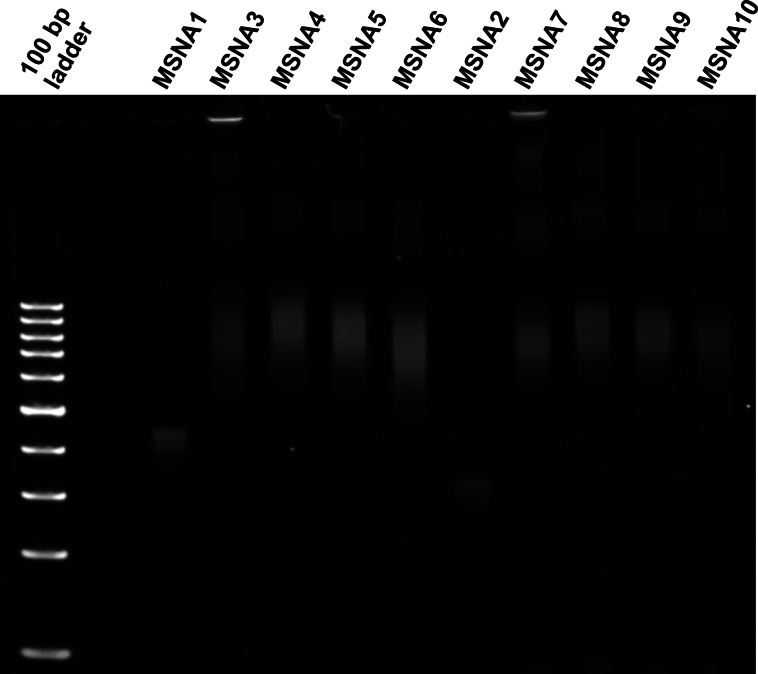
PAGE analysis of MSNAs. The ladder is only a reference and not suitable for determining the size of MSNAs. For conditions, see experimental section. (Note: With the given conditions, the excess oligonucleotide is eluted outside of the gel electropherogram)

**Figure 3:**
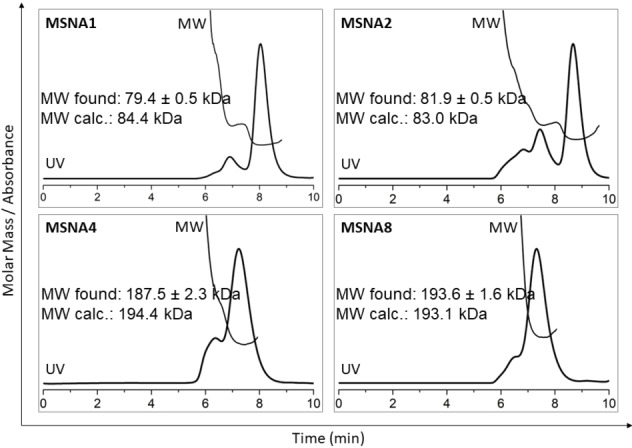
SEC‐MALS analysis of MSNAs. For conditions, see experimental section

[60]fullerene has been shown to be a valuable precursor for MSNAs. Its reaction with bis(azidoalkyl) malonates via Bingel's cyclopropanation^[54]^ offers a radially symmetric 12‐valent polyazide core (**4**) that can be conjugated with oligonucleotides using SPAAC to yield MSNAs.^[11]^ The density of oligonucleotides on **4** has been shown to be sufficient to activate scavenger A receptors and facilitate cellular uptake to breast cancer cells (MCF7), and down regulation of human epidermal growth factor receptor 2 (HER2) in ovarian cell line (SKOV3).^[11]^ Recently, we studied biodistribution properties of the [60]fullerene‐derived MSNAs *in vivo*,^[15]^ and demonstrated their potential *in vitro* as antibody‐conjugated^[12]^ and glyco‐coated drug delivery vehicles.^[14]^ Bingel's cyclopropanation with heteroalkyl malonates offers heterofunctional [60]fullerene‐derived core units, which can be used for the synthesis miktoarm MSNAs.[^13^, ^17^, ^18^] These MSNAs have been applied as scaffolds for abiotic intracellular catalysts^[17]^ and enzyme‐resistant nanoflares,^[18]^ and as more sophisticated gene regulation agents.^[18]^ Due to the encouraging previous results, 12‐valent polyazide core (**4**) was used for the synthesis of MSNAs in the present study (Scheme 2): 5′‐BCN‐modified 2′‐deoxy oligoribonucleotide **ON9** and oligoribonucleotide **ON10** were synthesized on a 1 μmol scale using an automatic DNA/RNA synthesizer and commercially available phosphoramidite building blocks. **ON9** and **ON10** were attached to azide‐modified [60]fullerene core **4** using SPAAC, following the procedure reported previously by our group.^[16]^ Because of solubility issues, the C_60_ core (**4**) was first conjugated with a sub‐stoichiometric amount (0.3 equivalents) of **ON9** or **ON10** in a mixture of DMSO and water. Thereafter, the obtained intermediate product (Scheme S1, Figures S8 and S9, Table 1) was treated with an excess of the same oligonucleotide in aqueous solution and in high salt concentration. This two‐step process alleviated the solubility issues and produced more homogenous MSNAs in 23 % (**MSNA1**) and 14 % (**MSNA2**) overall isolated yields (over two steps), purified by RP HPLC (Figure S10). Homogeneity, authenticity and hydrodynamic size of the MSNAs were verified by polyacrylamide gel electrophoresis (PAGE), size exclusion chromatography equipped with a multi angle light scattering detector (SEC‐MALS) and dynamic light scattering (DLS), respectively (cf. Figure 2, Figure 3 and Table 2).

**Table 1:** UV‐Thermal melting temperatures of the MSNAs.

	*T_m_*/°C
**MSNA3**	63.7±0.3
**MSNA4**	63.7±0.3 (−0.2)
**MSNA5**	63.6±0.7 (−0.1)
**MSNA6**	63.3±0.4 (−0.4)
**MSNA7**	73.5±0.2
**MSNA8**	73.6±0.6 (+0.1)
**MSNA9**	74.2±0.4 (+0.7)
**MSNA10**	73.0±0.5 (−0.5)

*ΔT_m_* values in parentheses are compared to either **MSNA3** (**MSNA4**–**6**) or to **MSNA7** (**MSNA8**–**10**). For conditions, see experimental section.

**Table 2:** Hydrodynamic size of the MSNAs.

	hydrodynamic size /nm
**MSNA1**	7.8±0.5
**MSNA3**	13.2±1.9
**MSNA4**	12.7±0.7
**MSNA5**	12.4±1.2
**MSNA6**	12.4±1.5
**MSNA2**	7.0±1.2
**MSNA7**	12.9±1.8
**MSNA8**	12.2±0.4
**MSNA9**	12.7±0.8
**MSNA10**	12.5±0.8

Conditions: 0.2 μmol L^−1^ MSNA+12 equivalents (2.4 μmol L^−1^) of the oligonucleotides (**ON5**–**8**) dissolved in 100 μL of PBS.

### Hybridization with Complementary MSNA

The hybridization of the oligonucleotide conjugates **ON5**–**8** with **MSNA1** and **MSNA2** was verified by UV thermal melting temperature analysis and PAGE. The melting temperature analysis was performed in 10 mmol L^−1^ sodium cacodylate buffer (pH 7.0) with 0.1 mol L^−1^ NaCl using 0.083 μmol L^−1^ the MSNA and 12 equivalents (1.0 μmol L^−1^) of the oligonucleotide. UV melting profiles of the oligonucleotide/**MSNA1** complexes (**MSNA3**–**6**) showed inflection points at 63–64 °C and of the oligonucleotide/**MSNA2** complexes (**MSNA7**–**10**) at 73–74 °C. The heteroduplexes on the RNA carrier (**MSNA2**) were ca Δ*T_m_* =10 °C more stable than the heteroduplexes on the DNA‐carrier (**MSNA1**). In each case the melting temperatures were high enough to convince the stability of the complexes at physiological temperature (Table 1). The impact of the CS moiety on the thermal stability of the oligonucleotide/MSNA complexes was marginal (|Δ*T_m_* |≤0.7 °C), as seen by comparing the melting temperatures of **MSNA4**–**6** (*T*_m_=63.3–63.7 °C) and **MSNA8**–**10** (*T*_m_=73.0–73.6 °C) to those of **MSNA3** (*T*_m_=63.7 °C) and **MSNA7** (*T*_m_=73.5 °C), respectively.

PAGE also confirmed the hybridization (Figure 2). To allow the hybridization, the samples were prepared in phosphate‐buffered saline (PBS), pH 7.4, before loading on the gel. **MSNA1** and **MSNA2** alone showed a distinct and relatively sharp band on gel. In both cases, a faster eluting byproduct, most probably an 11‐armed MSNA,^[16]^ was also detected. Nevertheless, **MSNA1** and **MSNA2** proved relatively pure after single RP‐HPLC purification (Figure S9). Hybridization of the MSNAs with **ON5**–**8** gave rise to a slower eluting and broad band on the gel, referring to formation of hybridization complexes **MSNA3**–**10**. Some even slower moving bands, likely caused by aggregation, were also observed. The bands of the hybridization complexes remained unchanged even if higher excess of oligonucleotides were used for the hybridization. A slight contribution of the CS decoration to the elution of the complexes could be seen.

Further characterization of MSNAs was performed by SEC‐MALS and DLS. Representative SEC‐MALS profiles (**MSNA1**, **MSNA2** and **MSNA4** and **MSNA8**) are shown in Figure 3. Each chromatogram shows a faster eluting peak, which refers to partial aggregation of the MSNAs that was also observed on PAGE (slower eluting bands). Molecular weights (MW) of the MSNAs, extracted from MALS of the major fraction matched well with the calculated ones, including also masses for the hybridization complexes (an average refractive index increment, dn/dc of 0.1703 mL g^−1^ used). In fact, the MALS‐based molecular weight was more accurate for the hybridization complexes than for the carrier MSNAs: **MSNA1**: MW_found_ 79.4±0.5 kDa (MW_calcd_ 84.4 kDa), **MSNA2**: MW_found_ 81.9±0.5 kDa (MW_calcd_ 83.0 kDa), **MSNA4**: MW_found_ 187.5±2.3 kDa (MW_calcd_ 194.4 kDa), **MSNA8**: MW_found_ 193.6±1.6 kDa (MW_calcd_ 193.1 kDa). As seen, the difference between the observed and calculated molecular weights was less than the mass of a single oligonucleotide, verifying complete decoration and hybridization of the MSNAs. For the DLS measurements, samples were prepared in PBS (pH 7.4) and hydrodynamic size of the MSNAs was determined. For the carriers: **MSNA1** and **MSNA2**, hydrodynamic size of 7.8 and 7.0 nm were observed. As expected, the hybridization with **ON5**–**8** increased the size. For **MSNA3**–**6**: 12.4–13.2 nm and for **MSNA7**–**10**: 12.2–12.9 nm were observed.

### Cellular Uptake and Intracellular Activity of the MSNAs

**Figure 4:**
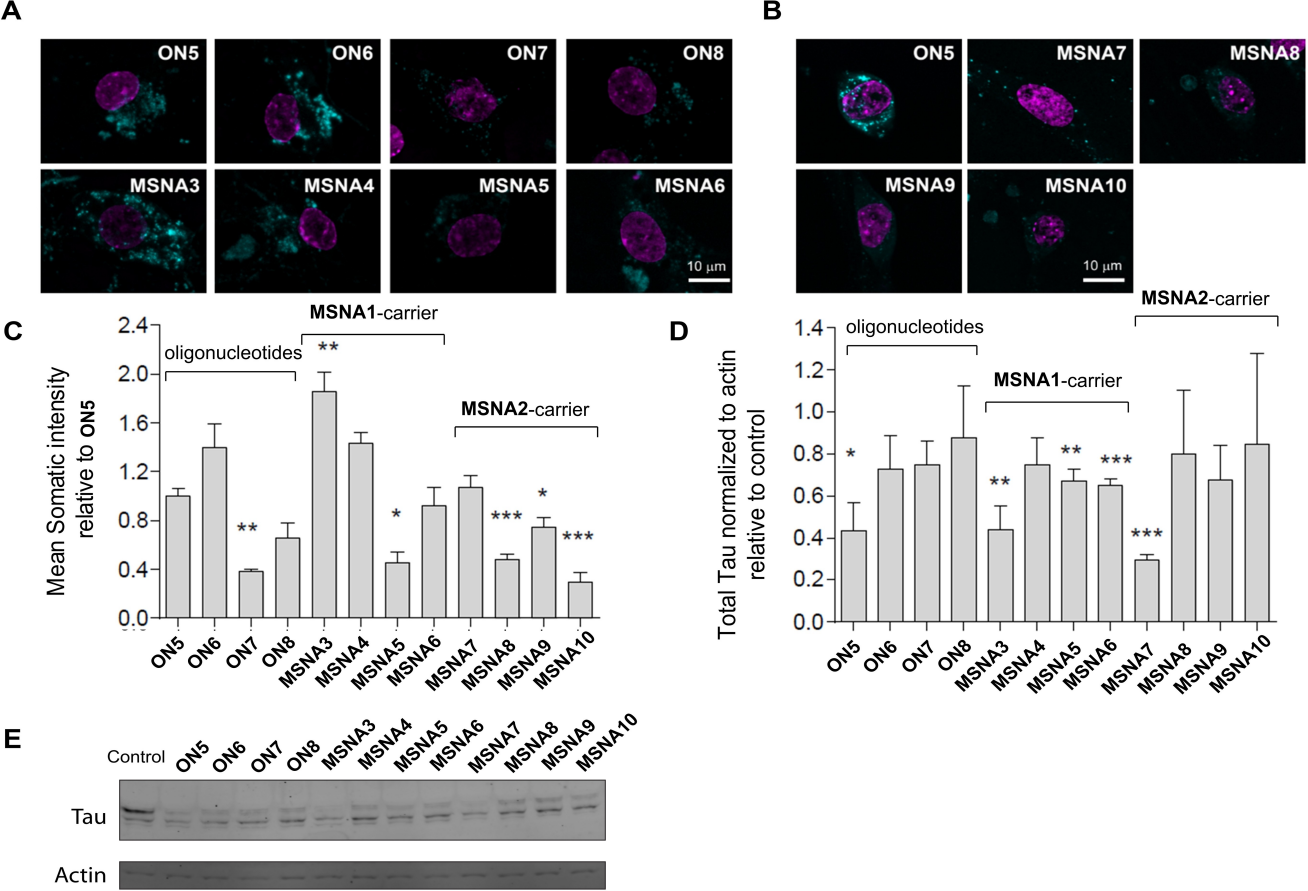
Uptake of **ON5**–**8** and **MSNA3**–**10** in neurons. A–B) Maximum intensity projections of five Z‐sections of 0.4 μm each through nuclei of hippocampal neurons treated at 7 days in culture with 120 nM **ON5**–**8** and 10 nM **MSNA3**–**10** for 10 days are shown (the effective ASO concentration of 120 nM in each case). Signal for the conjugates is shown in cyan while nuclei are highlighted in magenta. Scale=10 μm. C) Mean somatic intensity of AF488 signal relative to **ON5** from 3–6 cells +/− S.E.M for each conjugate is shown. The signal intensity refers to intact molecules (fluorescence quenching on MSNAs considered). D) Total Tau levels relative to actin and normalized to untreated cells analyzed by western blotting of Hippocampal neurons treated with indicated ASO conjugates and MSNAs as in A–B. Mean data +/− S.E.M from 3 repeats is shown. Significance was determined using Student's t‐test. *p<0.05; **p<0.01; ***p<0.001. (E) Representative images for treatment in D are shown.

Hippocampal neurons were prepared from rats and incubated for 10 days in culture with 120 nM solutions of anti‐Tau‐oligonucleotides (**ON5**–**ON8**) and 10 nM solutions of the corresponding MSNA‐heteroduplexes (**MSNA3**–**10**). Thus, the effective Tau‐ASO concentration in the experiments was the same (i. e. 120 nM). The obtained uptake data (Figure 4C) is comparable between the oligonucleotide conjugates (**ON5**–**ON8**) and between the hybridization complexes, i. e. **MSNA3**–**6** and **MSNA7**–**10**, but comparison between the conjugates and MSNAs can be confounded by different dissociation/degradation rates and fluorescence quenching on the MSNAs. The signal intensity refers to intact molecules, in which fluorescence quenching (Figure S11) on MSNAs has been considered. By examining the oligonucleotides (**ON5**–**ON8**) themselves, it could be seen that the CS moiety and its sulfation pattern had impact on the uptake. The CS‐tetrasaccharide with R^1^=H and R^2^=SO_3_^−^ (**ON6**, cf. Scheme 1) increased the uptake, whereas the other two CS variants (**ON7** and **ON8**, cf. Scheme 1) decreased the uptake, compared to **ON5**. The deep understanding how different CS structures affect the uptake needs further studies. However, the sulfate groups more on the outer sphere (**ON7** and **ON8** vs **ON6**) seemed to have a negative effect on the uptake, which may suggest to shift the interest more towards hyaluronic‐like structures (i. e. non‐sulfated glycans). The DNA‐MSNA‐carrier increased the uptake (**MSNA3**), whereas the uptake by RNA‐MSNA carrier (**MSNA7**) remained at a similar level compared to **ON5**. However, the aforementioned difference between the concentrations (10 nM vs. 120 nM) of the complexes and of the oligonucleotides, as well as the potential interference in fluorescence intensity, should be considered in this evaluation. While the CS‐conjugate group could show some beneficial effect on the uptake of oligonucleotides (**ON6** vs **ON5**), similar effect could not be found on CS‐coated MSNAs (**MSNA4**–**6** and **MSNA8**–**10**). However, the uptakes of CS‐decorated **MSNA4**–**6** followed the same trend as observed with Tau‐ASO‐conjugates **ON6**‐**ON8**. The effect of CS‐decoration on the uptake remained modest, considering previous findings with neurons and CS‐coated gold nanoparticles,^[44]^ but may still encourage evaluation of other CS structures as decorates of MSNAs. The CS decoration studied in the present study probably interferes the scavenger receptor A‐mediated cellular uptake of the MSNAs, which cannot be compensated by beneficial CS‐ligand‐driven effect on the uptake, if any with hippocampal neurons (cf. **ON6** *vs*. **ON5**). After the uptake experiments, the treated neurons were lyzed and introduced to Western blot assay. Total Tau level was compared to actine and normalized to untreated cells (Figures 4D and E). A marked Tau suppression could be observed in cells treated with **ON5**, **MSNA3** (i. e. **ON5** on the DNA‐MSNA carrier) and **MSNA7** (i. e. **ON5** on the RNA‐MSNA carrier). While the most effective Tau‐suppression could be observed with **MSNA7**, no clear influence caused by the chemistry of MSNAs (i. e. “DNA vs RNA carrier”) was observed. These results are positive and may encourage for further studies evaluating the concept of heteroduplex ASOs[^33^, ^34^] on MSNAs. The CS conjugate group on **ON6**–**8** (and CS‐decoration on **MSNA4**–**6** and **MSNA8**–**10**) interfered with Tau suppression. This was somewhat expected within these heavily‐modified demo Tau‐ASO‐sequences, the primary goal of which was to evaluate the impact of CS‐decoration on the cellular uptake of MSNAs. Modifications at both terminus of the ASO are likely to interfere RNase‐H‐mediated cleavage of the mRNA target.

### Enzymatic Stability of MSNAs

To verify the enzyme‐mediated cleavage of MSNAs, they were incubated with DNase I and RNase H, and the potential degradation of the MSNAs and simultaneous release of the Tau‐ASOs were monitored by PAGE (Figure S12). **MSNAs3**–**6** (on the DNA carrier, **MSNA1**) underwent rapid and complete DNase 1‐catalyzed degradation (1 U DNase I/nmol of the effective oligonucleotide content, 6 h at 37 °C), whereas **MSNAs7**–**10** (on the RNA carrier, **MSNA2**) remained mainly intact even by a prolonged incubation in the same conditions (10 U DNase I/nmol of the effective oligonucleotide content, 3 d at 37 °C). **MSNAs7**–**10**, fulfilling better the concept of heteroduplex ASOs, in turn, proved substrates for RNase H (10 U RNase H/nmol of the effective oligonucleotide content, 3 d at 37 °C). However, the degradation of the RNA‐carrier (**MSNA2**) proved relatively slow (c.a. 60 % degradation of **MSNA7** after 3 d) and it seemed to be interfered on CS‐decorated **MSNAs8**–**10**. The data obtained by these enzyme experiments were partly in line with the Western blot assay above. RNase H‐activity of the Tau mRNA‐ASO‐complex is likely to be the limiting step for the down regulation of Tau protein, which can be interfered by CS‐moieties on **ON6**‐**ON8**. In each case, the enzyme‐catalyzed (DNase I or RNase H) degradation of the carrier (**MSNA1** or **MSNA2**) released intact Tau‐ASOs (**ON5**‐**ON8**) as monitored by PAGE (Figure S12).

## Conclusions

The applicability of [60]fullerene‐based molecular spherical nucleic acids (MSNAs) as hybridization‐mediated carriers (DNA‐ and RNA‐based carriers, **MSNA1** and **MSNA2**) for an antisense oligonucleotide, suppressing Tau protein, (i. e. Tau‐ASO) and its conjugates with chondroitin sulfate tetrasaccharides (CS) with different sulfation patterns (three different variants) was demonstrated. For the characterization of the obtained heteroduplex Tau‐ASO‐MSNAs, PAGE, SEC‐MALS, UV‐melting profile analysis and DLS were applied. SEC‐MALS proved a valuable tool, which offered molecular mass analysis for the hybridization‐mediated complexes of ca. 190 kDa. The hippocampal neuron uptake and activity suppressing Tau protein of the CS‐Tau‐ASO‐conjugates (**ON6**–**ON7**) and of the corresponding heteroduplex MSNAs (**MSNA4**–**6** and **MSNA8**–**10**) were studied in vitro by confocal microscopy and Western blot analysis, respectively. The impact of the MSNA carriers, CS‐moieties on the conjugates and the CS‐decorations on the MSNAs were evaluated. One of the CS‐Tau‐ASO‐conjugates enhanced the uptake to neuron cells (**ON6**), as did the DNA‐based heteroduplex MSNA carrier (**MSNA3**), but the other two CS‐ASO‐conjugates and MSNA‐formulations of the conjugates had negative or marginal effect on the uptake. These results, while modest, may still prompt to evaluate other CS halos on the MSNAs. Previous findings with CS‐coated nanoparticles have been encouraging, and imply that the neuronal uptake is sensitive to the CS structure.^[44]^ A significant suppression of Tau protein could be observed by Western blot assay in neuron cells treated with Tau‐ASO without CS‐conjugation (**ON5**) and its heteroduplexes on DNA‐ and RNA‐based MSNAs (**MSNA3** and **MSNA7**). The suppression of Tau protein was interfered by CS conjugation on oligonucleotides (**ON6**–**ON8**) and on the corresponding MSNAs (**MSNAs4**–**6** and **MSNAs8**–**10**). This finding was supported by enzymatic stability tests (DNase I and RNase H) of MSNAs. Accordingly, RNase H‐activity of the Tau mRNA‐ASO‐complex may be the limiting step of the down regulation, which was interfered by CS‐moieties on **ON6**–**ON8**. Despite the interfering effect of the CS conjugation with these model compounds, the results are encouraging and may prompt for further studies with more potent ligand decoration. By applying the concept of heteroduplex ASO on MSNAs, making them substrates for RNase H, may open new possibilities for MSNA‐derived drug delivery vehicle design.

## Experimental Section

### General Remarks

RP‐HPLC analysis and purification of the oligonucleotide conjugates was performed by an analytical RP C18 (250×4.6 mm, 5 μm) column unless stated otherwise. A flow rate of 1.0 ml/min, a detection wavelength of 260 nm and gradient A, B or C were employed. Gradient A: 0–70 % MeCN in 0.1 M aqueous triethylammonium acetate (pH 7) over 25 min. Gradient B: 0–95 % MeCN in 0.1 M aquous triethylammonium acetate (pH 7) over 25 min. Gradient C: 40–100 % MeCN in 50 mM aquous triethylammonium acetate (pH 7) over 20 min The mass spectra were recorded using either MS (orbitrap) or MS (ESI‐TOF) spectrometer. The isolated yields of the oligonucleotide conjugates were determined according to their UV absorbance at 260 nm.

### Synthesis of Oligonucleotide Conjugates

5′‐BCN‐modified oligonucleotides **ON1**, **ON9** and **ON10** were synthesized on a 1.0 μmol scale using an automatic DNA/RNA synthesizer. Commercially available solid supports (including the 3′‐aminomodifier) and phosphoramidite building blocks of 2′‐deoxy, 2′‐*O*‐(2‐methoxyethyl) and 2′‐O‐TBDMS nucleosides, a 5′‐amino modifier and 2‐(bicyclo[6.1.0]non‐4‐yn‐9yl)ethan‐1‐ol were used for the assembly. **ON1**, **ON9** and **ON10** were released from the supports by the usual ammonolysis protocol (followed by TBDMS removal in case of **ON10**) and homogenized by RP‐HPLC using gradient A.

CS‐oligonucleotides conjugates **ON2**–**4** were synthesized as follows (Scheme 1): **ON1** (88 nmol in 27 μL H₂O) was combined with azide‐modified CS tetrasaccharide **1** (263 nmol in 128 μL of H₂O). The reaction was incubated at room temperature overnight and the resulting conjugate **ON2** was purified by RP‐HPLC using gradient A (Figure S1). The HPLC fractions were collected and lyophilized to dryness to yield 19 nmol (22 %) of **ON2**. **ON3** and **ON4** were synthesized following the same procedure in 18 % and 22 % yields, respectively. The authenticity of the products was verified by MS (ESI‐TOF, Table S1, Figures S1–S3).

AF488‐labelling of the oligonucleotides was performed as follows: **ON2** (19 nmol) was lyophilized to dryness and dissolved in 20 μL of sodium borate buffer (0.1 mol L^−1^, pH 8.4). AF488 NHS ester (380 nmol in 2 μL DMSO) was added, the reaction was incubated at room temperature overnight, and the resulting oligonucleotide **ON6** was purified by RP‐HPLC using gradient B. The HPLC fractions were collected and lyophilized to dryness to yield 17 nmol (88 %) of **ON6**. **ON5**, **ON7** and **ON8** were prepared by the same protocol. Isolated yields varied between 85 % and 91 % (Table S1). The authenticity of the products was verified by MS (ESI‐TOF, Table S1, Figures S4‐S7).

### Synthesis of MSNA1 and MSNA2

To a solution of **4** (120 nmol in 130 μL DMSO), 5′‐BCN‐modified oligonucleotide **ON9** (40 nmol in 20 μL H_2_O) was added. The reaction mixture was gently shaken overnight at room temperature and introduced to RP‐HPLC using gradient C (Figure S9). The HPLC fractions were collected and lyophilized to dryness to yield 19 nmol (47 %) of the monofunctionalized [60]fullerene‐oligonucleotide intermediate product [MS (ESI‐TOF), Figure S9]. The intermediate product (9.0 nmol) was mixed with **ON9** (130 nmol, 1.2 equivalents of **ON9** / azide arm) in 1.5 M aqueous NaCl solution and the reaction mixture was gently shaken for 72 h at room temperature. The resulting **MSNA1** was purified by RP‐HPLC using an analytical Phenomenex Aeris WIDEPORE XB−C18 200 Å (150×4.6 mm, 3.6 μm) column with a linear gradient of 5–45 % MeCN in 50 mM aqueous triethylammonium acetate (pH 7) over 30 min (Figure S10). The HPLC fractions were collected and lyophilized to dryness to yield 4.4 nmol (48 %) of **MSNA1**. A slightly modified procedure was used for the synthesis of **MSNA2** (cf. supporting information), obtained in 14 % overall isolated yield (over the two steps). The homogeneity and authenticity of **MSNA1** and **MSNA2** was confirmed by PAGE and SEC‐MALS (cf. Figure 1 and Figure 3). The hydrodynamic sizes of **MSNA1** and **MSNA2** were determined by DLS (cf. Table 2).

### PAGE Analysis of MSNAs

Native 6 % Tris base, boric acid, ethylenediaminetetraacetic acid (EDTA), and acrylamide (TBE) gel were used for assessing the purity of the MSNAs and their hybridized complexes with oligonucleotides **ON5**–**8**. A pre‐cast gel cover (10 cm ×10 cm in size, Thermo Fisher Scientific) was fixed into a vertical electrophoresis chamber, and the running buffer (90 mM Tris, 90 mM borate, and 2 mM EDTA, pH 8.3) was filled into the chamber. Samples were prepared by mixing 2 μl of 0.05 μM MSNA in PBS (pH 7.4) with 2 μl of TBE sample buffer. For the hybridization complexes (**MSNA3**‐**MSNA10**), 24 equivalents (1.2 μM, corresponding to a 2‐fold excess) of the complementary oligonucleotides **ON5**–**8** were used. The samples were loaded onto the gel and electrophoresed at constant 100 V for 50 min. After completion, the gel was removed from the chamber and stained with SYBR^TM^ gold Nucleic Acid Stain (Thermo Fisher Scientific). Each gel was run with a 100 bp ladder in one of the wells.

### Melting Temperature Analysis of MSNAs

The thermal melting curves of the hybridized MSNA/oligonucleotide complexes were measured at 260 nm with a PerkinElmer Lambda 35 UV‐vis spectrometer equipped with a multiple cell holder and a Peltier temperature controller. The measurements were performed in 10 mmol L^−1^ sodium cacodylate buffer (pH 7.0) in 0.1 M aqueous NaCl. MSNA concentration in each sample was 0.083 μmol L^−1^ and 12 equivalents (1.0 μmol L^−1^) of complementary oligonucleotides were used. The temperature was changed at a rate of 0.5 °C/min (10–90 °C). Each sample was heated up and cooled down three times and *T_m_*‐values were determined as the maxima of the first derivatives of the melting curves.

### DLS Experiments

The hydrodynamic size of the MSNAs were measured using a Zetasizer Nano ZS90 (Malvern Instruments Ltd. UK). The measurements were performed at room temperature in PBS (pH 7.4). The MSNA concentration in each sample was 0.2 μmol L^−1^ and 12 equivalents (2.4 μmol L^−1^) of complementary oligonucleotides **ON5**–**8** were used in the hybridized samples. Each sample was measured six times and the hydrodynamic diameter was determined as the average of the measurements.

### SEC‐MALS Experiments

SEC‐MALS was performed using a 1260 Infinity II HPLC system (sampler, pump, and UV‐VIS detector; Agilent Technologies, Santa Barbara, CA, USA) equipped with a miniDAWN light scattering detector and Optilab refractive index detector (Wyatt Technologies, Santa Barbara, CA, USA). An Advance Bio SEC 300 Å 2.7 μm 4.6×300 mm column (Agilent, Santa Clara, CA, USA) and 150 mM sodium phosphate, pH 7.0, as mobile phase eluting at a rate of 0.2 mL min^−1^ and run time of 20 min were used for each experiment. For each run, 40 μL of MSNA sample (0.08–0.16 μg/μl) was loaded onto a pre‐equilibrated column. The refractive index was used for the molecular weight calculations using an average refractive index increment (dn/dc) of 0.1703 mL/g.

### Primary Neuronal Cultures and Confocal Imaging

Hippocampal neurons were prepared from rats as described previously.^[55]^ At 7 days in vitro, neurons were treated with 120 nM of the indicated conjugates and 10 nM of the MSNAs for 10 days (the effective ASO concentration of 120 nM in each case). Cells were washed with PBS and fixed with 4 % cold paraformaldehyde in phosphate buffered saline and stained with Hoechst‐33342 (1 : 2000). Z‐sections of 0.4 μm through nuclei of 17 day in vitro hippocampal neurons were obtained using Zeiss 880 LSM using the 63X oil objective (1.4 NA) using 488 nm (2.3 % laser power) for conjugates or 405 nm laser lines (18 % laser power) for Hoechst‐33342. Scan used 2‐line averaging with minimum pixel dwell of 0.24 μs and pixel size of 0.06 μm×0.06 μm. Maximum intensity projections of 5 sections through nuclei were generated using the software Zen from Zeiss and the mean somatic intensities were measured using Fiji.

### Western Blotting

Hippocampal neurons treated with conjugates and MSNAs as above were lyzed in 1x Laemmli buffer supplemented with 1 mM MgCl2, 50 U/ml Benzonase, 1× Roche cOmplete, Mini, protease inhibitor cocktail and 1× Phosphatase inhibitor cocktail 1 (P2850, Sigma). Lysates were resolved on 10 % SDS PAGE gels. Membranes were probed with Tau (#4019, Cell Signalling Technology) and Actin (#A3853, Sigma‐Aldrich). IRDye® 680RD Donkey anti‐Mouse IgG Secondary Antibody (#926‐68072, Licor) was used to detect the signal and images were acquired using the Odyssey western blot imager. Densitometry was performed using the image analysis software Fiji.

### Enzymatic Stability Tests

10 mM tris‐HCl (pH 7.5) with 2.5 mM MgCl_2_, 0.1 mM CaCl_2_ and 4 mM NaCl was used for DNase I experiments, and 20 mM tris‐HCl (pH 7.8) with 40 mM KCl, 8 mM MgCl_2_, 1 mM dithiotreitol and 4 mM NaCl for RNase H experiments. **MSNAs3**–**6** were treated with DNase I (1 U/nmol of the effective oligonucleotide content). **MSNAs7**–**10** were treated with DNase I (10 U/nmol of the effective oligonucleotide content) and RNase H (10 U/nmol of the oligonucleotide content). The solutions (18.8 μL containing 38 pmol of oligonucleotide) were incubated at 37 °C and 2 μL samples were taken at appropriate time intervals. The samples were added into TBE sample buffer (2 μL) on ice and analyzed by gel electrophoresis (6 % TBE acrylamide gel electrophoresed at constant 100 V for 50 min) (Figure S12). The extent of **MSNA7** degradation was quantified by the image analysis software Fiji.

## Supporting Information

Synthesis of **MSNA2**, RP HPLC profiles and MS spectra of the oligonucleotide conjugates **ON2**–**ON4**, **ON5**–**ON8**, RP HPLC profiles of **MSNA1** and **MSNA2** and fluorescence quenching on MSNAs and PAGE analysis of the enzymatic stability experiments are described in the Supporting Information.

## Conflict of Interests

The authors declare no conflict of interest.

1

## Supporting information

As a service to our authors and readers, this journal provides supporting information supplied by the authors. Such materials are peer reviewed and may be re‐organized for online delivery, but are not copy‐edited or typeset. Technical support issues arising from supporting information (other than missing files) should be addressed to the authors.

Supporting Information

## Data Availability

The data that support the findings of this study are available from the corresponding author upon reasonable request.
