# Supplementary material for: Chondroitin Sulfate‐Coated Heteroduplex‐Molecular Spherical Nucleic Acids
Source: Chembiochem. 2024 Nov 28;26(6):e202400908. doi: 10.1002/cbic.202400908 (PMC11907394; doi:10.1002/cbic.202400908)
Supplement: Supplementary file 1 — Supporting Information [file CBIC-26-e202400908-s001.pdf]

ChemBioChem

Supporting Information

Chondroitin Sulfate-Coated Heteroduplex-Molecular Spherical Nucleic Acids

Toni Laine, Prasannakumar Deshpande, Ville Tähtinen, Eleanor T. Coffey, and Pasi Virta*

Supporting Information to

Chondroitin Sulfate-Coated Heteroduplex-Molecular Spherical Nucleic Acids

Toni Laine[a], Prasannakumar Deshpande[b], Ville Tähtinen[a], Eleanor T. Coffey[b], Pasi Virta[*,a]

[a] T. Laine, Dr. V. Tähtinen, Prof. P. Virta
Department of Chemistry
University of Turku
20500 Turku, Finland
E-mail: pamavi@utu.fi

[b] Dr. P. Deshpande, Prof. E. T. Coffey
Turku Bioscience Centre
University of Turku, Åbo Akademi University
20520 Turku, Finland

CONTENTS

Synthesis of **MSNA2** S2

MS data and isolated yields of oligonucleotide conjugates (**ON2-ON4**, **ON5-ON8**, **S1** and **S2**) (Table 1) S3

RP-HPLC profiles and mass spectra of oligonucleotide conjugates (**ON2-ON4**, **ON5-ON8**, **S1** and **S2**) S4-S8

RP-HPLC profiles of **MSNA1** and **MSNA2** S9

Fluorescence quenching on the MSNA S9

MSNAs treated with DNase I and RNase H S10

Synthesis of MSNA2

C_{60}-fullerene core **4** (30 nmol in 180 µL DMSO) was added to a solution of **ON10** (10 nmol in 24 µL H_2O). The reaction was incubated at room temperature overnight and the monosubstituted product **S2** was purified by RP-HPLC using an analytical C18 column (250 × 4.6, 5µm) with a linear gradient from 40–100 % MeCN in aqueous 50 mM triethylammonium acetate (pH 7). A flow rate of 1.0 ml/min and a detection wavelength of 260 nm were employed (Figure S10). After lyophilization, **S2** was dissolved in nuclease free water and characterized by MS (ESI-TOF). The isolated yield (3.4 nmol, 34 %) was determined UV-spectrometrically. **S2** (2.3 nmol in 32 µL of H_2O) and **ON10** (41 nmol in 235 µL of H_2O) were combined and lyophilized together. The residue was dissolved in 54 µL of 0.75 M NaCl and the reaction was gently shaken at room temperature for 72 h. The resulting **MSNA2** was purified by RP-HPLC using an analytical Phenomenex Aeris WIDEPORE XB-C18 200 Å (150 × 4.6 mm, 3.6 µm) column with a linear gradient of 5–45 % MeCN in 50 mM aqueous triethylammonium acetate (pH 7) over 30 min. A flow rate of 1.0 ml min^{-1} and a detection wavelength of 260 nm were employed. After lyophilization, **MSNA2** was dissolved in water and the isolated yield (0.96 nmol, 42 %) was determined UV-spectrometrically.

Scheme S1. Synthesis of MSNAs.

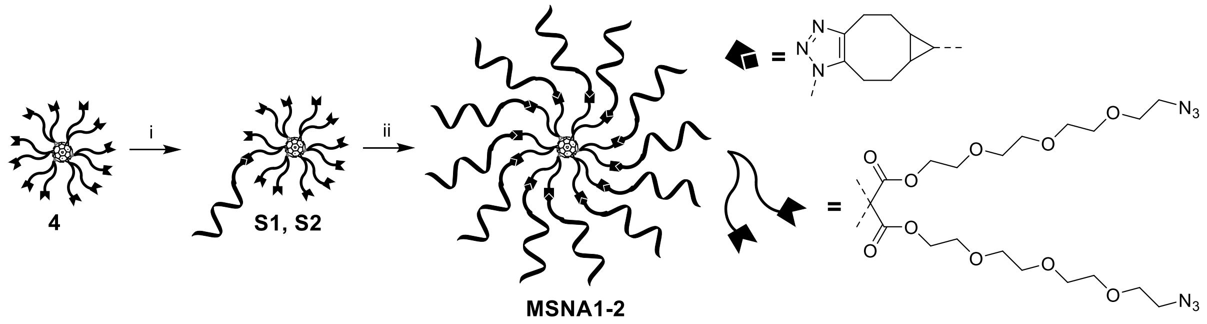

Notes: C_{60}-based core is monosubstituted with **ON9** or **ON10** to yield compounds **S1** and **S2,** respectively. **S1** and **S2** are treated with an excess of **ON9** or **ON10** to yield **MSNA1** and **MSNA2.** Conditions: i) **ON9** or **ON10** (0.3 equivalents / **4**), DMSO:H_2O (9:1, *v/v*), overnight at room temperature; ii) excess **ON9** or **ON10** in 0.75M aqueous NaCl, 3 d at room temperature

Table S1. MS data and isolated yields of oligonucleotide conjugates

Oligonucleotide-conjugate	Observed mass	Calculated mass	Isolated yield / %
**ON2** [a]	8659.8	8659.4	22
**ON3** [a]	8659.4	8659.4	18
**ON4** [a]	8818.9	8818.7	22
**ON5** [b]	8150.1198	8150.1201	85
**ON6** [b]	9169.3362	9169.3063	88
**ON7** [b]	9169.3414	9169.3063	85
**ON8** [b]	9329.2482	9329.2199	91
**S1** [a]	10469.1	10469.2	47
**S2** [a]	10532.7	10531.9	34

[a] The observed and calculated molecular mass values are reported according to average masses. The observed masses are calculated based on the most intensive isotope combination (ESI-TOF). [b] The observed and calculated molecular mass values are reported according to monoisotopic masses (orbitrap).

RP-HPLC profiles and mass spectra of oligonucleotide conjugates

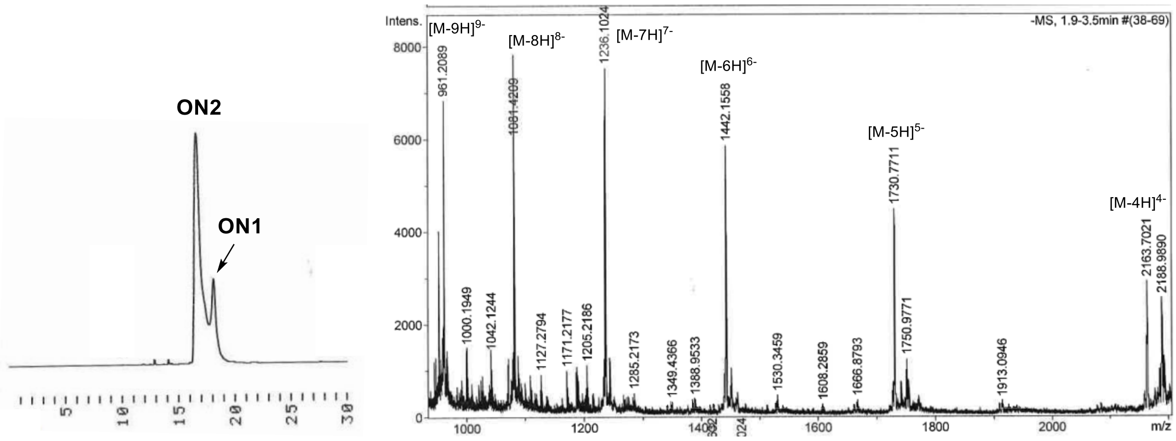

Figure S1. A) Crude RP-HPLC profile and B) MS (ESI-TOF) spectrum of **ON2**. RP-HPLC conditions: An analytical C18 column (250 × 4.6 mm, 5 µm) with a linear gradient of 0–70 % MeCN (0.1 M triethylammonium acetate, pH 7) over 25 min, detection at λ = 260 nm, flow rate 1.0 mL min^{-1}.

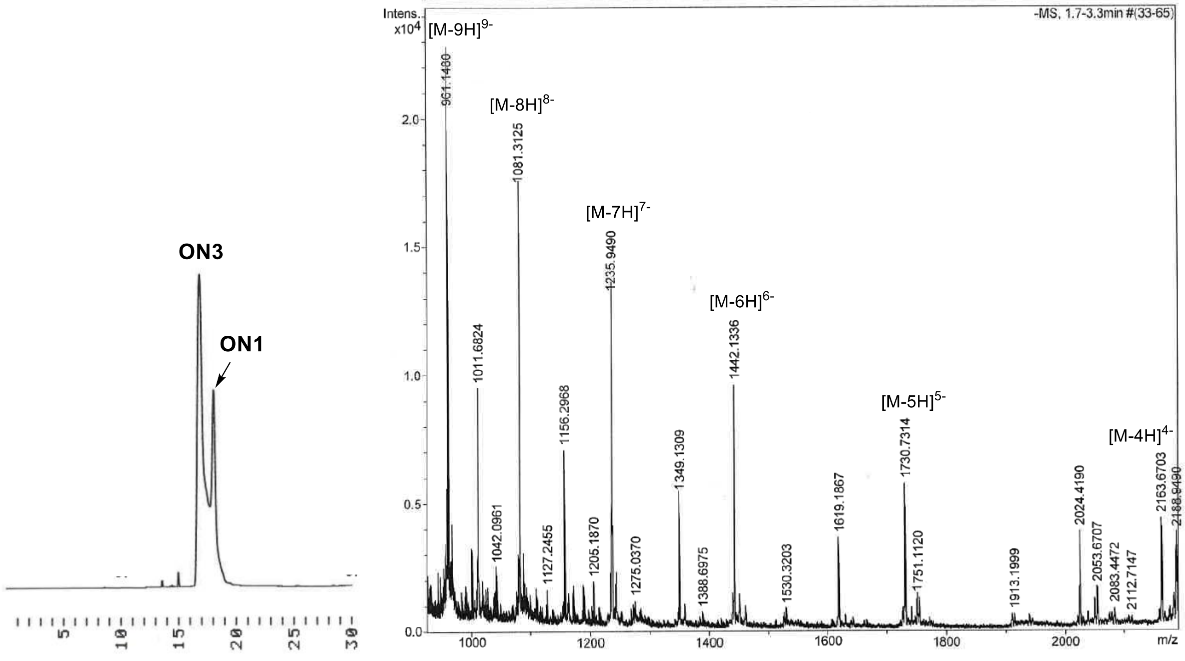

Figure S2. A) Crude RP-HPLC profile and B) MS (ESI-TOF) spectrum of **ON3**. RP-HPLC conditions: An analytical C18 column (250 × 4.6 mm, 5 µm) with a linear gradient of 0–70 % MeCN (0.1 M triethylammonium acetate, pH 7) over 25 min, detection at λ = 260 nm, flow rate 1.0 mL min^{-1}.

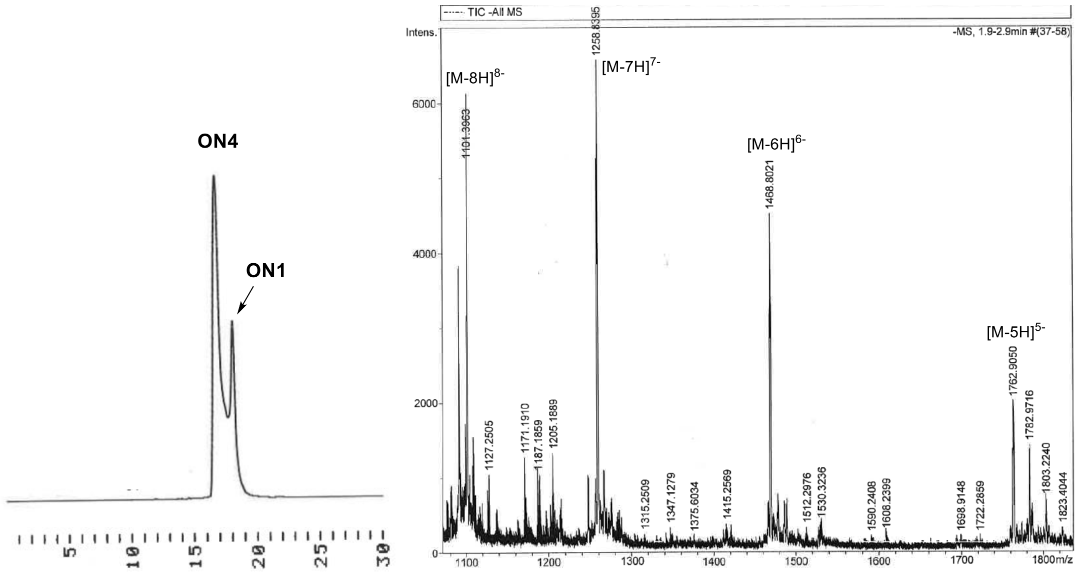

Figure S3. A) Crude RP-HPLC profile and MS (ESI-TOF) spectrum of **ON4**. RP-HPLC conditions: An analytical C18 column (250 × 4.6 mm, 5 μm) with a linear gradient of 0–70 % MeCN (0.1 M triethylammonium acetate, pH 7) over 25 min, detection at λ = 260 nm, flow rate 1.0 mL min^{-1}.

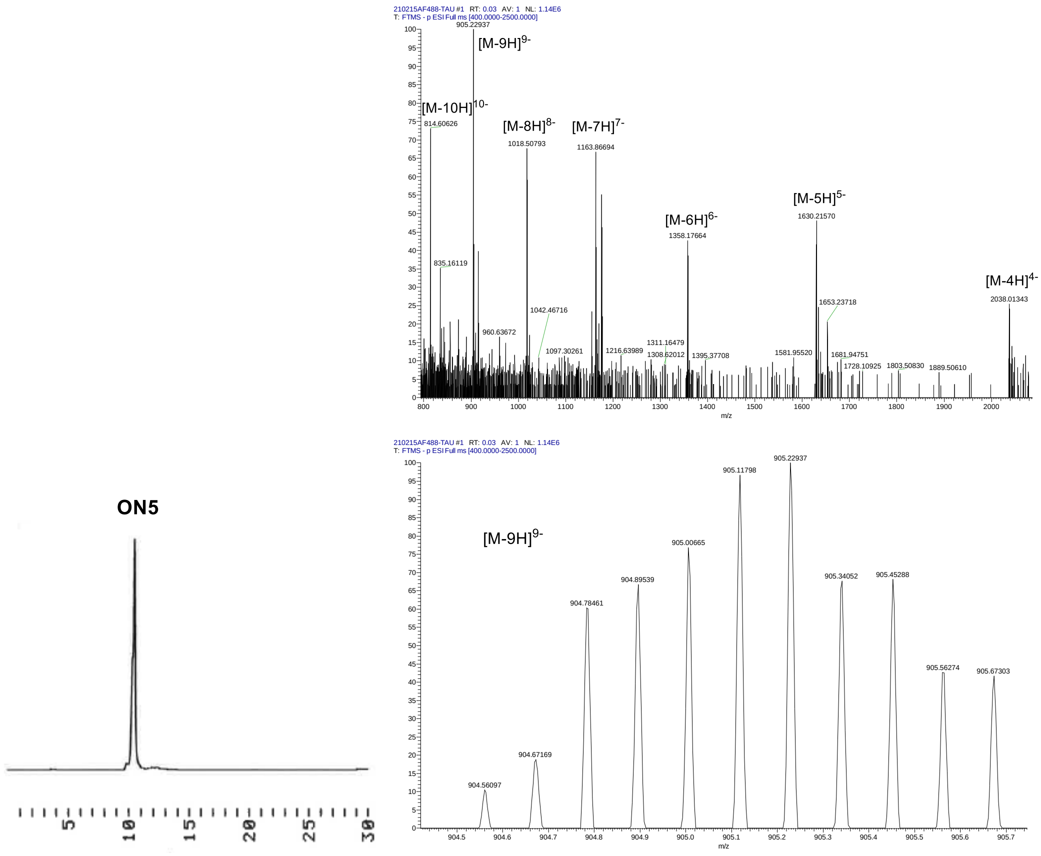

Figure S4. A) RP-HPLC profile and B) MS (orbitrap) spectrum of homogenized **ON5**. RP-HPLC conditions: An analytical C18 column (250 × 4.6 mm, 5 μm) with a linear gradient of 5–95 % MeCN (0.1 M triethylammonium acetate, pH 7) over 25 min, detection at λ = 260 nm, flow rate 1.0 mL min^{-1}.

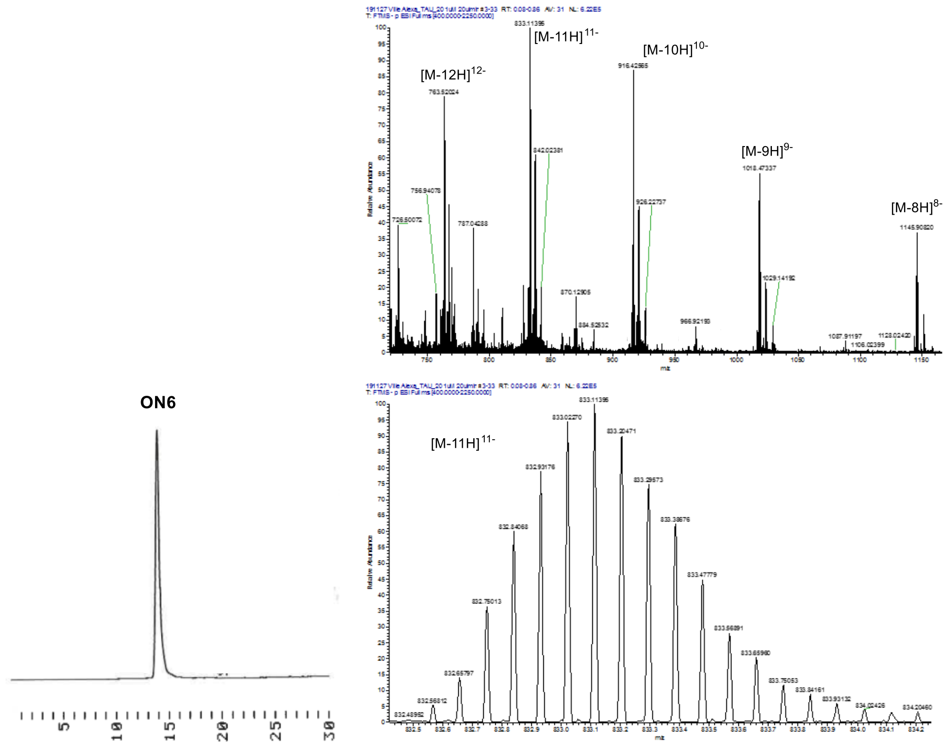

Figure S5. A) RP-HPLC profile and B) MS (orbitrap) spectrum of homogenized **ON6**. RP-HPLC conditions: An analytical C18 column (250 × 4.6 mm, 5 µm) with a linear gradient of 5–95 % MeCN (0.1 M triethylammonium acetate, pH 7) over 25 min, detection at λ = 260 nm, flow rate 1.0 mL min^{-1}.

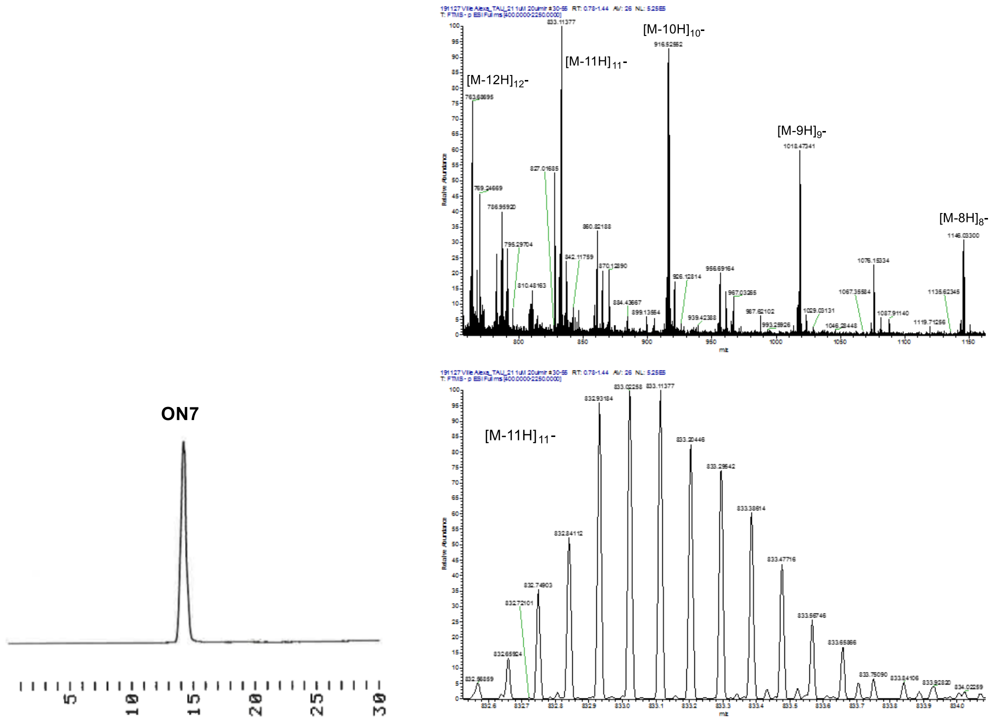

Figure S6. A) RP-HPLC profile and B) MS (orbitrap) spectrum of homogenized **ON7**. RP-HPLC conditions: An analytical C18 column (250 × 4.6 mm, 5 µm) with a linear gradient of 5–95 % MeCN (0.1 M triethylammonium acetate, pH 7) over 25 min, detection at λ = 260 nm, flow rate 1.0 mL min^{-1}.

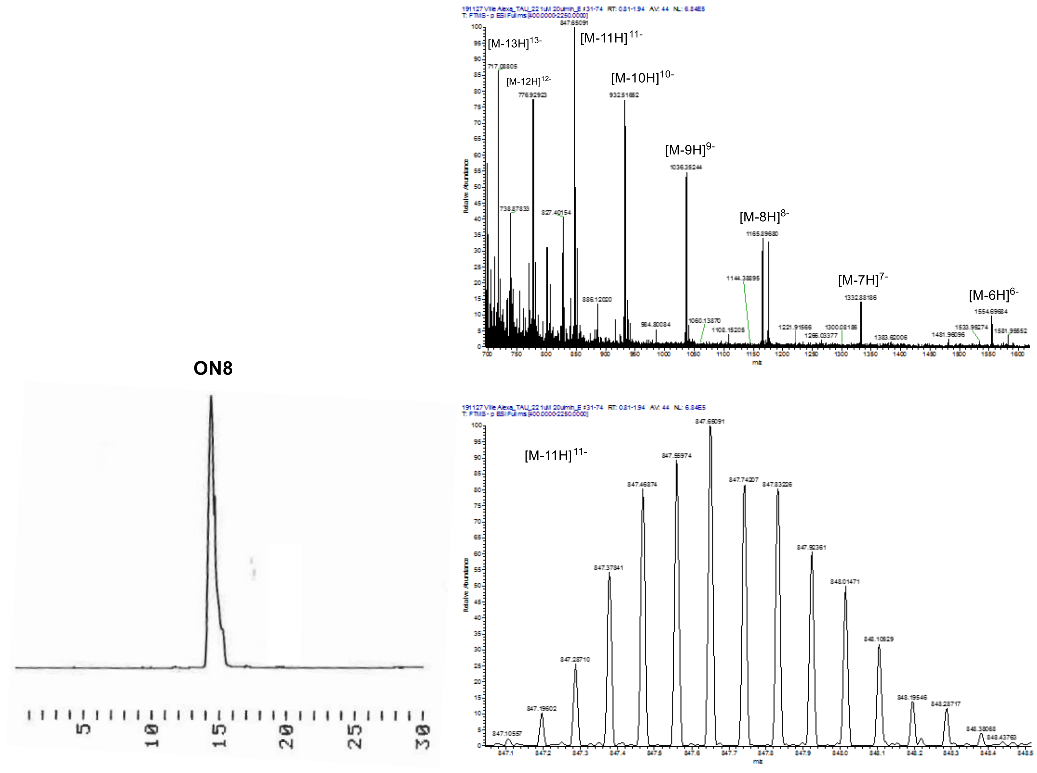

Figure S7. A) RP-HPLC profile and B) MS (orbitrap) spectrum of homogenized **ON8**. RP-HPLC conditions: An analytical C18 column (250 × 4.6 mm, 5 µm) with a linear gradient of 5–95 % MeCN (0.1 M triethylammonium acetate, pH 7) over 25 min, detection at λ = 260 nm, flow rate 1.0 mL min^{-1}.

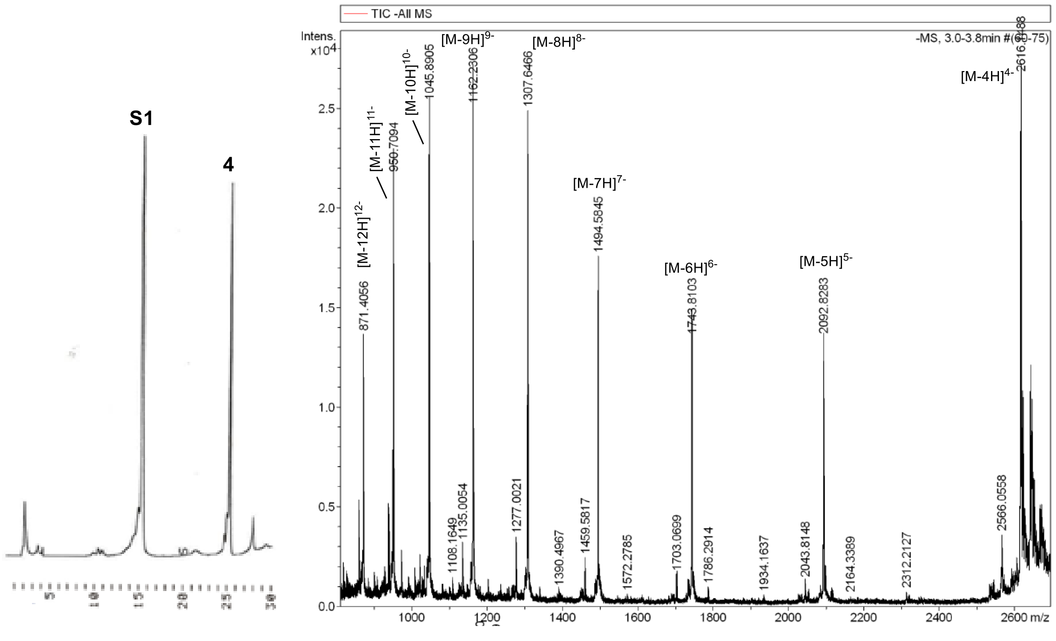

Figure S8. A) Crude RP-HPLC profile and B) MS (ESI-TOF) spectrum of **S1**. RP-HPLC conditions: An analytical C18 column (250 × 4.6 mm, 5 µm) with a linear gradient of 40–100 % MeCN (50 mM triethylammonium acetate, pH 7) over 20 min followed by 100 % MeCN from 20–30 min, detection at λ = 260 nm, flow rate 1.0 mL min^{-1}.

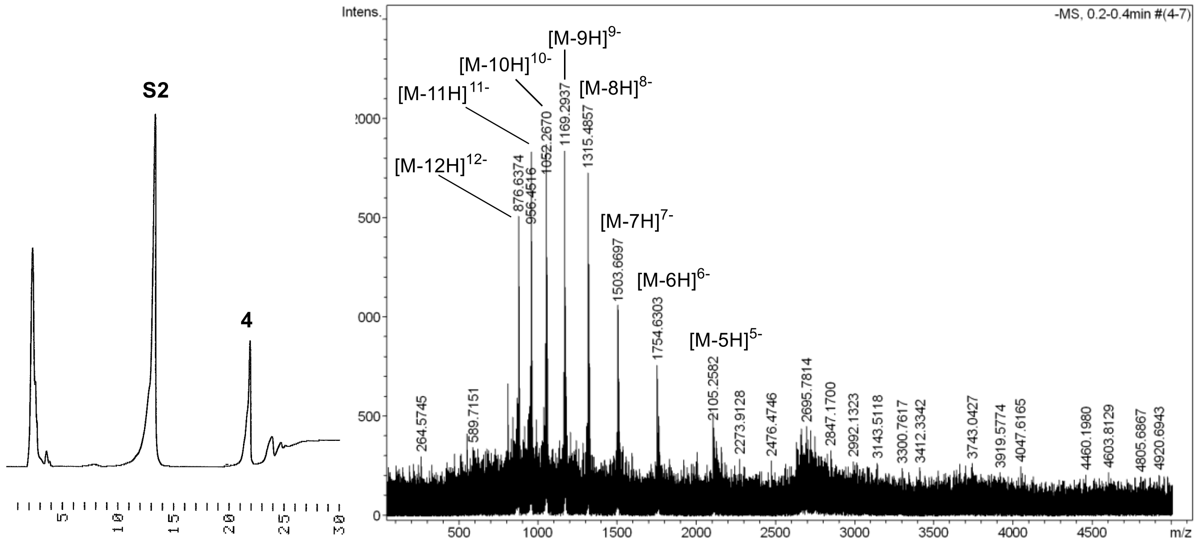

Figure S9. A) Crude RP-HPLC profile and B) MS (ESI-TOF) spectrum of **S2**. RP-HPLC conditions: An analytical C18 column (250 × 4.6 mm, 5 µm) with a linear gradient of 40–100 % MeCN (50 mM triethylammonium acetate, pH 7) over 20 min followed by 100 % MeCN from 20–30 min, detection at λ = 260 nm, flow rate 1.0 mL min^{-1}.

Crude RP-HPLC profiles of MSNA1 and MSNA2

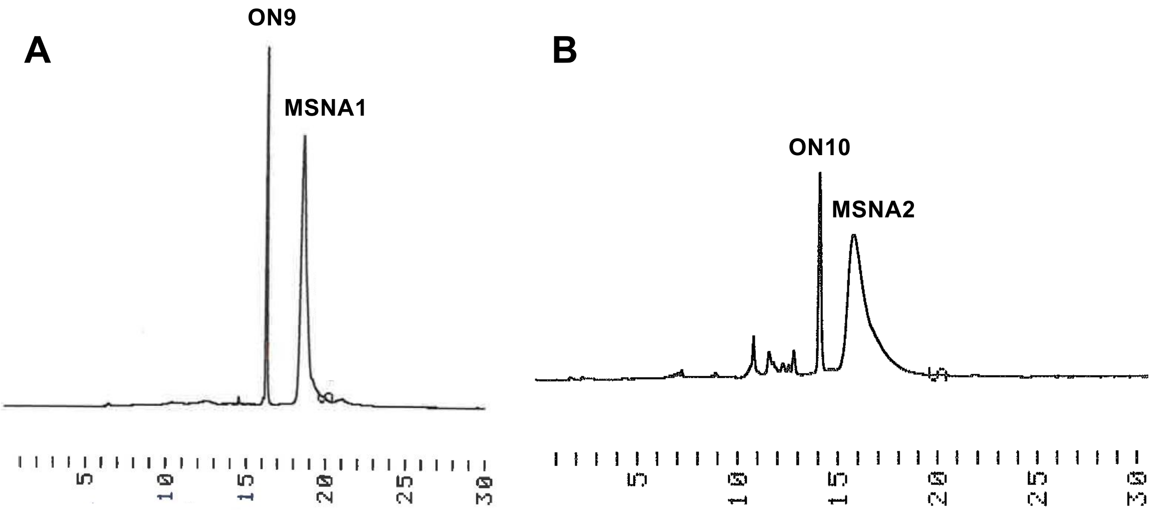

Figure S10. Crude RP-HPLC profiles of A) **MSNA1** and B) **MSNA2**. RP-HPLC conditions: Phenomenex Aeris WIDEPORE XB-C18 200 Å (150 × 4.6 mm, 3.6 µm) column with a linear gradient of 5–45 % MeCN (50 mM triethylammonium acetate, pH 7) over 30 min, detection at λ = 260 nm, flow rate 1.0 mL min^{-1}.

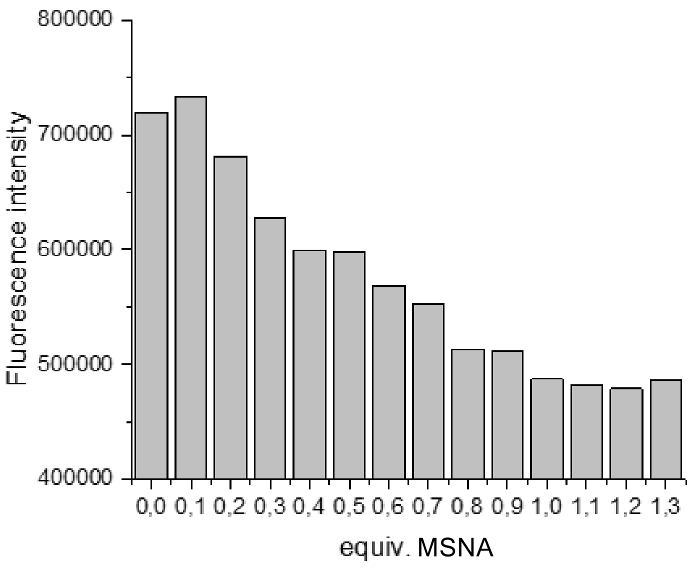

Figure S11 demonstrates how much duplex formation on the MSNA quenches the fluorescence intensity of the AF488-label. **ON8** was titrated with **MSNA1** in PBS (pH 7.4) at room temperature. The concentration of **ON8** was 0.5 µmol L^{-1} and the **MSNA1** was added in 0.1 equiv. aliquots (refers to effective concentration of the oligonucleotide strands). After each addition, a fluorescence spectrum was recorded. The samples were excited with λ = 495 nm and the fluorescence at λ = 519 nm was recorded. As the amount of MSNA increased, a decrease in fluorescence was observed. No further quenching was observed after reaching 1.0 equiv. of MSNA. The fully hybridized structure quenched the fluorescence by 33 % compared to free **ON8**.

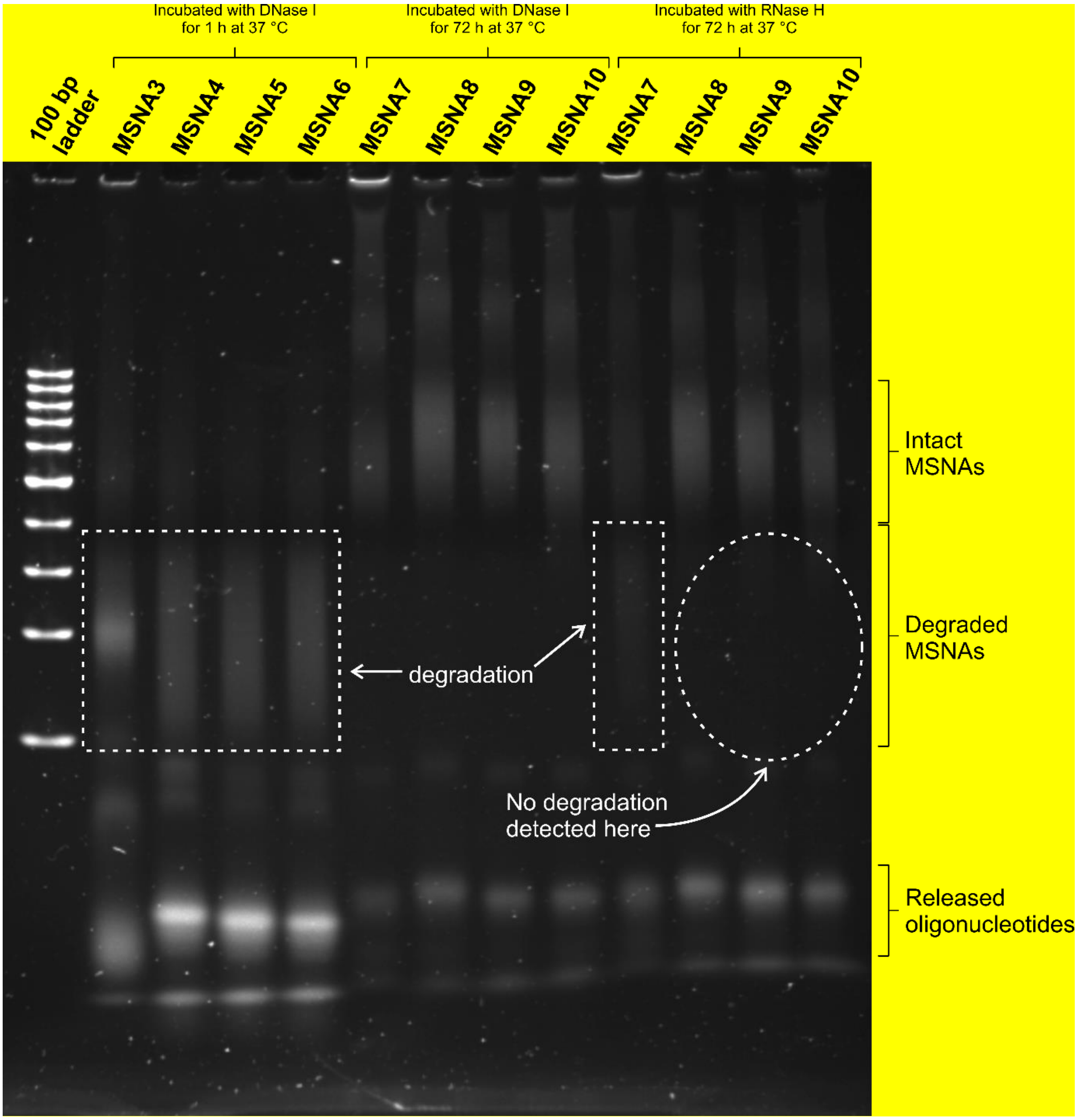

Figure S12. MSNAs treated with DNase I and RNase H. Conditions: 10 mM tris-HCl (pH 7.5) with 2.5 mM $MgCl_2$, 0.1 mM $CaCl_2$ and 4 mM NaCl was used for DNase I experiments. 20 mM tris-HCl (pH 7.8) with 40 mM KCl, 8 mM $MgCl_2$, 1 mM dithiotreitol and 4 mM NaCl for RNase H experiments. **MSNAs3–6** were treated with DNase I (1 U/nmol of the effective oligonucleotide content). **MSNAs7–10** were treated with DNase I (10 U/nmol of the effective oligonucleotide content) and RNase H (10 U/nmol of the effective oligonucleotide content).
